# Small Bowel Obstruction Due to Transperitoneal Incarceration After Anterior Lumbar Interbody Fusion: A Case Report

**DOI:** 10.7759/cureus.67214

**Published:** 2024-08-19

**Authors:** Arizona Binst, Joost Maurissen, Karl De Pooter, Eva Vangenechten, Paul Storms

**Affiliations:** 1 General and Abdominal Surgery, St-Elisabeth General Hospital Herentals, Herentals, BEL

**Keywords:** abdominal hernia repair, bowel incarceration, anterior lumbar inter-body fusion, preperitoneal hernia, small bowel herniation, small-bowel obstruction

## Abstract

We present the case of a 76-year-old woman who experienced severe abdominal pain and vomiting 14 days after undergoing anterior lumbar interbody fusion. CT revealed a mechanical small bowel obstruction with a transition point in the proximal ileum. During surgery, an incarcerated bowel loop was discovered, having herniated into the preperitoneal space. The obstruction was corrected surgically. This article discusses the perioperative findings, reviews the existing literature, and examines surgical correction techniques. We emphasize the importance of meticulous peritoneal closure and the need for vigilance against intra-abdominal complications in retroperitoneal surgical procedures.

## Introduction

Anterior lumbar interbody fusion (ALIF) is a surgical technique used for lumbar spine fusion through anterior extraperitoneal access. This approach is associated with a shorter operating time and reduced perioperative morbidity compared to other techniques. Common indications for ALIF include spondylolisthesis, degenerative disc disease, degenerative lumbar scoliosis, pseudoarthrosis, and adjacent segment disease [1].

To the best of our knowledge, this case is only the second reported instance of a retro/extraperitoneal hernia resulting in intestinal obstruction following anterior retroperitoneal spinal exposure.

## Case presentation

We present the case of a 76-year-old female patient who arrived at our regional ED with severe abdominal pain and vomiting that persisted for five days. Her medical history revealed an ALIF procedure performed 14 days earlier at another hospital. She had no other abdominal surgical history or significant comorbidities. The ALIF procedure and an eight-day hospital stay had been uneventful.

The patient initially visited our ED two days prior with an inability to pass stools for eight days. A plain abdominal X-ray showed fecal matter in the colon and diffuse air-fluid levels in the small bowel. After receiving an enema, she produced stools successfully and was discharged with oral osmotic laxatives, diagnosed with transient subobstruction.

**Figure 1:**
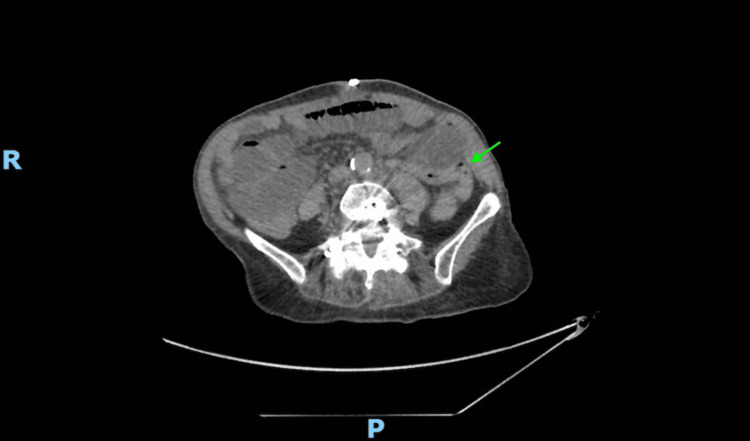
Axial view of CT of the abdomen showing dilated small bowel loops with angulation of the bowel loops in the left iliac fossa (green arrow)

Upon her return to the ED two days later, a CT scan of the abdomen revealed a mechanical small bowel obstruction with a transition point at the proximal ileum (Figure 1). Conservative treatment with nasogastric decompression failed, leading to an exploratory laparoscopy four days after admission.

**Figure 2:**
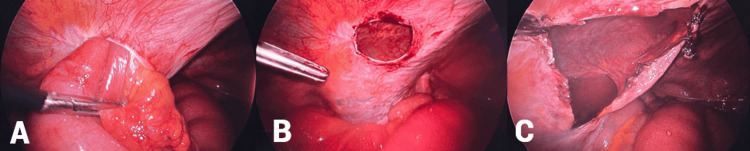
Per operative images during laparoscopy (A) Herniated small bowel loop through the peritoneum in the left iliac fossa. (B) Remaining defect in the peritoneum after reduction of the herniated bowel loop. (C) Incised peritoneum.

 A 5 mm trocar was inserted in the epigastrium and left iliac fossa. During surgery, an opening in the parietal peritoneum was identified, through which a loop of small bowel had herniated into the preperitoneal Bogros’ space (Figure 2A). A recent resorbable suture at the defect’s border suggested incomplete closure of the peritoneal breach from the ALIF procedure two weeks prior. The herniated bowel was laparoscopically reduced without bowel resection. Due to the rigidity of the parietal peritoneum, we chose to further incise it to prevent recurrent incarceration (Figure 2B, 2C). A flexible silicone drain was placed to prevent fluid accumulation.

The postoperative course was uneventful, with smooth re-alimentation and normal bowel function. The drain was removed on postoperative day two, and the patient was discharged on postoperative day four.

## Discussion

A manual literature search on PubMed identified only one other case of transperitoneal herniation following retroperitoneal lumbar surgery [2]. Colosimo et al. described a patient who, six months postoperatively, presented with symptoms of obstruction. The patient had been admitted twice previously for intestinal obstruction, which was managed conservatively. On this occasion, an emergency laparotomy revealed an incarcerated hernia in the retroperitoneal space. The retroperitoneum was incised, and the bowel was reduced, but there is no mention of whether the defect was closed.

Multiple similar cases have been reported following laparoscopic total extraperitoneal inguinal hernia repair. The pathophysiology of these cases is similar. During primary surgery, accidental disruption of the parietal peritoneum can occur during extraperitoneal dissection. An attempt is usually made to close this disruption; however, an inadequately closed defect may enlarge, allowing the bowel to herniate into the newly dissected preperitoneal space. The intraoperative manipulation of the typically elastic parietal peritoneum can stiffen it due to an inflammatory process, creating a mechanical barrier between the peritoneal cavity and the preperitoneal space and forming a potential hernia site.

The timing of presentation ranges from one day to three weeks postoperatively [3-7]. In all cases, the peritoneal defect was closed using either resorbable or non-resorbable sutures, with one case using resorbable sutures reinforced with 2 ml of fibrin sealant [3]. The peritoneal defect, often discovered during explorative laparoscopy or laparotomy, is typically closed after reducing the herniated bowel. In our case, the parietal peritoneum had become too large and rigid to close effectively. Therefore, the defect was incised further to prevent recurrent incarceration (Figure 2C).

Common complications of ALIF include vascular damage, neurological injury, retrograde ejaculation, surgical site infection, incisional hernias, and postoperative ileus [1,5,6]. The incidence of peritoneal disruption during ALIF is reported to be between 0.14% and 0.44% [7]. Meticulous closure of incidental peritoneal defects with resorbable sutures and vigilant inspection for accidental disruptions are crucial, as demonstrated in our case. Employing an access surgeon, such as an experienced vascular or abdominal surgeon for spinal exposure, may help limit complications; however, studies show conflicting results regarding the benefit of incorporating access surgeons in teams with adequately trained spinal surgeons [7]. Maintaining a high index of suspicion for intra-abdominal complications in extraperitoneal surgery is essential. Postoperative symptoms such as nausea, vomiting, failure to pass stools for several days, or a worsening recurrence of symptoms should prompt further investigation.

## Conclusions

This report underscores the critical importance of meticulous peritoneal closure during retroperitoneal surgery, regardless of whether the surgeon specializes in abdominal or non-abdominal procedures. Additionally, it emphasizes the necessity for physicians to remain vigilant for intra-abdominal complications following such retroperitoneal surgeries.
